# The exploration of medical resources utilization among inguinal hernia repair in Taiwan diagnosis-related groups

**DOI:** 10.1186/s12913-017-2665-6

**Published:** 2017-11-09

**Authors:** Yu-Hua Yan, Chih-Ming Kung, Yi Chen

**Affiliations:** 1 Department of Medical Research, Tainan Municipal Hospital (Managed by Show Chwan Medical Care Corporation), No. 670, Chungde Rd, East Dist, Tainan City, 70173 Taiwan, Republic of China; 2 Department of Hospital and Health Care Administration, Chia Nan University of Pharmacy and Science, No.60, Sec. 1, Erren Rd., Rende Dist, Tainan City, 71710 Taiwan, Republic of China; 3 Department of Information Technology and Communication, Shih Chien University Kaohsiung Campus, 200 University Road Neimen, Kaohsiung City, 84550 Taiwan, Republic of China; 4 Superintendent Office, Tainan Municipal Hospital (Managed by Show Chwan Medical Care Corporation), No. 670, Chungde Rd, East Dist, Tainan City, 70173 Taiwan, Republic of China

**Keywords:** Taiwan diagnosis-related groups (Tw-DRGs), Inguinal hernia repair, Resource utilization, Global budgeting system (GBS)

## Abstract

**Background:**

This study centered on differences in medical costs, using the Taiwan diagnosis-related groups (Tw-DRGs) on medical resource utilization in inguinal hernia repair (IHR) in hospitals with different ownership to provide suitable reference information for hospital administrators.

**Methods:**

The 2010–2011 data for three hospitals under different ownership were extracted from the Taiwan National Health Insurance claims database. A retrospective method was applied to analyze the age, sex, length of stay, diagnosis and surgical procedure code, and the change in financial risk of medical costs in IHR cases after introduction of Tw-DRGs. The study calculated the cost using Tw-DRG payment principles, and compared it with estimated inpatient medical costs calculated using the fee-for-service policy.

**Results:**

There were 723 IHR cases satisfying the Tw-DRGs criteria. Cost control in the medical care corporation hospital (US$764.2/case) was more efficient than that in the public hospital (US$902.7/case) or nonprofit proprietary hospital (US$817.1/case) surveyed in this study. For IHR, anesthesiologists in the public hospital preferred to use general anesthesia (86%), while those in the two other hospitals tended to administer spinal anesthesia. We also discovered the difference in anesthesia cost was high, at US$80.2/case on average.

**Conclusions:**

Because the Tw-DRG-based reimbursement system produces varying hospital costs, hospital administrators should establish a financial risk assessment system as early as possible to improve healthcare quality and financial management efficiency. This would then benefit the hospital, patient, and Bureau of National Health Insurance.

## Background

Taiwan’s National Health Insurance (NHI) scheme was launched in 1995, and since 1998 its revenue from premiums has fallen short of medical expenditure. In July 1998, the Bureau of National Health Insurance (BNHI) initiated a global budgeting system (GBS) for dental treatment to help control medical expenses. The BNHI subsequently extended the GBS to traditional Chinese medicine treatment in July 2000, and hospital care in July 2002. The NHI uses both retrospective and prospective payment systems (RPS and PPS, respectively). Originally, the unit of payment was mainly fee-for-service, although case payment was used for certain diagnoses, and per diem payment was also allowed for chronic psychiatric problems and community services. Despite various legislative and administrative measures aimed at capping maximum reimbursement, including a global budget system and eventually a case-payment scheme, the rapid increase of medical expenses continued. The BNHI began using a Taiwan-specific diagnosis-related group system (Tw-DRG) in January 2010. Every service is accompanied by a co-payment, irrespective of the patient’s age [1, 2].

The Medicare payment policy has also changed over time, including changes in the diagnosis-related group (DRG) codes assigned to different constellations of the International Classification of Disease, Ninth Revision, Clinical Modification (ICD-9-CM) diagnoses and procedures, and the weights assigned to those DRGs [3]. The BNHI first introduced the Tw-DRGs for items with simple medical costs and few options to conserve medical resources and reduce financial pressure. Tw-DRGs divide patients into groups depending on diagnosis, surgical or treatment procedure, age, sex, existence of complications, length of stay (LoS), and their medical resource consumption. The payment for each group, of which there were several hundred, was determined in advance. Except for in special cases, hospitals receive the same payment for all patients in the same group [4].

In the first year of implementation, the BNHI added a total of 164 items to the Tw-DRGs [1, 2]. According to conservative estimates based on comparative populations, there are approximately 40,000 IHR cases annually in Taiwan. Hernias are among the oldest recorded human health afflictions, and IHR is the most common general surgical procedure for treating them [5–7]. A hernia is defined as a protrusion or projection of an organ, or a part of an organ, through the body wall that normally contains it. Inguinal hernia is more common than femoral hernia and other abdominal wall hernias (e.g., umbilical and epigastric hernia).

Hospital costs associated with the treatment include direct costs (e.g., mesh and supplies) and indirect costs (e.g., facility fees and equipment depreciation) [8]. Therefore, under the Tw-DRG system, hospital operators face uncertainty and the risk of financial responsibility, and healthcare professionals are required to control the medical cost to reduce wastage of medical resources. NHI system reform will change the concept of healthcare management practice from pursuit of profit to pursuit of efficiency. This is emerging in the changes to the incentive system; transitioning from an outcome-based contract to one based on behavior. Such adjustment is expected to change healthcare behaviors and operating procedures, and force hospitals to focus more on effectiveness and efficiency [9].

Hospitals in Taiwan can be categorized by ownership (public, private, or corporate), type of medical treatment provided (general, chronic disease, or psychiatric), education capability (teaching or non-teaching), and level of accreditation (medical center, regional hospital, or district hospital [10]). This study aimed to determine the differences in medical costs with the introduction of Tw-DRGs regarding medical resource utilization for IHR in hospitals under different forms of ownership. This would also provide reference information for hospital administrators.

## Methods

### Database

This was a retrospective study using convenience sampling. Data from 2010 to 2011 were extracted from the NHI claims database for three hospitals under different forms of ownership, all in the Show Chwan Healthcare System. This system included the public Tainan Municipal Hospital (543 beds), the medical care corporation Show Chwan Memorial Hospital (711 beds), and the nonprofit proprietary Chang Bing Show Chwan Memorial Hospital (1126 beds). All cases were selected from among the Tw-DRGs: Unilateral Inguinal and Femoral Hernia Procedures (DRG16202), and all patients were over 18 years old and had no complications or comorbidities. Operating room procedures included repair of inguinal hernia, direct inguinal hernia, indirect inguinal hernia, direct inguinal hernia with graft or prosthesis, indirect inguinal hernia with graft or prosthesis, inguinal hernia with graft or prosthesis, not otherwise specified, unilateral repair of femoral hernia with graft or prosthesis, and other unilateral femoral herniorrhaphy. Under the Tw-DRGs system, hospitals are paid a fixed fee to treat patients in a single DRG category, regardless of the actual costs. However, the NHI adjusted the payment price in 2011; therefore, the NHI fixed fee in 2010–2011 was US$934.5 and US$947.5 (exchange rate: US$1 = NT$30).

### Research variables and operational definitions

In Taiwan, any operations with ICD-9-CM primary surgical procedure codes of 53.00–53.05, 53.21, and 53.29 are reimbursed as IHR under the Tw-DRGs. We therefore compared the return on IHR hospitalization with a statistical analysis on the NHI claims data for the three hospitals. Table 1 shows the research variables and operational definitions.

**Table 1:** Study variables and operational definitions

Variable	Operational definition
Sex	Male or female.
Age	Based on the World Health Organization definition and divided into dependent and non-dependent populations for a total of four groups: teenagers, 18–24 years; young adults, 25–44 years; adults, 45–64 years; and older adults, 65 years or above.
Diagnosis code	Inpatient diagnosis based on the declaration of inpatient payment regulated by the Bureau of National Health Insurance (BNHI); one principal diagnosis and four secondary diagnoses max. Total number of diagnoses.
Procedure code	Procedure based on the declaration of inpatient payment regulated by the BNHI; one principal procedure and four procedures maximum. Total number of procedures.
DRG fixed cost (*R*)	1. Tw-DRGs fixed payment = relative weight (RW) × standard payment rate (SPR) × (one basic treatment weight + pediatric weight + CMI weight + hospitals in mountainous region and offshore island weight) 2. Verification declaration for the lower limit of payment 3. Upper limit of payment = Tw-DRGs fixed payment*0.8
Estimated medical cost (*R*_*1*_)	Total costs calculated by the corresponding NHI payment standard for treatment provided.
Cost difference (*∆R*)	Fixed cost, *R* – estimated medical cost, *R*_*1*_
Ratio of cost difference over fixed cost (*∆R/R*)	(Fixed cost, *R* – estimated medical cost, *R*_*1*_)/DRG fixed cost

We included sex and age (as hernia can occur at any age, and divided patients into four groups: those aged 18–24, 25–44, 45–64, and 65 and above. Experiences in the United States and other countries that have implemented DRGs suggest hospitals could earn more by increasing the number of cases with comorbidity and complications (CC) or by changing the diagnosis. The BNHI disease code requirements include one principal diagnosis and up to four secondary diagnoses. The number of diagnoses was calculated based on all cases. The number of surgical procedures is also subject to particular criteria under the BNHI, with one principal surgical procedure and up to four secondary procedures. Procedures per case was calculated as the total procedure coding numbers divided by the total number of cases. The LoS is the total number of calendar days between the inpatient admission and discharge, excluding the actual day of discharge. Patients admitted and discharged on the same day have a calculated LoS of 1 day.

Names of all medical cost items are based on the BNHI classification, including: diagnosis, ward, tube feeding, laboratory, X-rays, therapeutic procedures, blood/plasma, hemodialysis, special materials, drugs, dispensing services, and injection services. We used the cost calculated using the Tw-DRGs payment principles (i.e., fixed cost, R) and compared it with the estimated inpatient medical cost (R1) calculated using the fee-for-service policy. (ΔR) was the difference (R − R1) between the two costs. The ratio of cost difference over cost calculated using Tw-DRG payment principles (ΔR/R) was determined for each DRG. The value of ΔR/R represented the inpatient medical services return rate, which also represented the financial risk of treating DRG patients. Cost difference (ΔR) was treated as the financial return on providing the inpatient medical service. The ratio of this cost difference over fixed cost (ΔR/R) was viewed as the inpatient medical service premium or loss ratio.

### Statistical analysis

We used SPSS for Windows version 18.0 (SPSS Inc., Chicago, Illinois, USA) for all data analysis work. The descriptive statistics show the analysis of various variables including age, sex, LoS (days), number of diagnoses, and the number of procedures, showing the quantity, percentage, mean, and standard deviation. Using NHI inpatient declaration files, we statistically analyzed the returns earned by hospitals from inpatients. We used a t-test to examine differences in number of diagnoses, surgical treatments, complications and comorbidities, average LoS, and anesthesia statistics. Analysis of variance (ANOVA) was used to examine medical costs, and anesthesia LoS statistics. We defined *p* > 0.05 as not significant (ns).

## Results

Between 2010 and 2011, 723 IHR cases met the Tw-DRGs criteria, of which 315 were in the public hospital, 254 in the medical care corporation hospital, and 154 in the nonprofit proprietary hospital. Most patients were male: 88.9%, 92.1%, and 89.0% in the three hospitals, respectively (*p* > 0.01). The majority were also older people (aged 65 or above). The average age of IHR cases across all hospitals was 58 years. The average LoS was 1.97 days, mean number of the coding diagnoses was 1.66, and mean number of coding procedures was 1.04 (Table 2). We used a χ^2^ test to analyze the descriptive data.

**Table 2:** Descriptive statistics (*n* = 723)

Country	Public Hospital		Medical Care Corporation Hospital		Non-Profit Proprietary Hospital		Mean	SD	*p* ^*a*^
	*n*	%	*n*	%	*n*	%			
No. of patients	315		254		154				
Age	58		59		56		58.0	16.73	0.442
Age category 1: 18–24	8	2.5	8	3.1	5	3.2			
Age category 2: 25–44	54	17.1	38	15.0	36	23.4			
Age category 3: 45–64	131	41.6	101	39.8	54	35.1			
Age category 4: >65	122	38.7	107	42.1	59	38.3			
Sex									0.387
Male	280	88.9	234	92.1	137	89.0			
Female	35	11.1	20	7.9	17	11.0			
LoS (days)	2.6		1.3		1.7		1.97	1.01	<0.001
LoS 1 day	23	7.3	180	70.9	82	53.2			
LoS 2 days	114	36.2	58	22.8	45	29.2			
LoS 3 days	145	46.0	14	5.5	21	13.6			
LoS 4 days	29	9.2	2	0.8	3	1.9			
LoS 5 days	4	1.2	0	0.0	3	1.9			
No. diagnoses	1.6		1.7		1.5		1.66	0.94	<0.033
No. procedures	1.04		1.04		1.02		1.04	0.16	0.670

*SD* standard deviation

^a^There were significant differences (*P* < 0.001) in all variables of patients’ characteristics among enrollees, potential enrollees, and comparison groups. Statistical difference was calculated using a *x* ^2^ test for categorical variables and a t-test for continuous variables

The average cost per case of the medical care corporation hospital (US$764.2/case) suggests it practices more efficient cost control than the public hospital (US$902.7/case) or nonprofit proprietary hospital (US$817.1/case) (*p* < 0.001). Using the difference between the Tw-DRGs reimbursement and estimated medical cost as a measure of cost control, the nonprofit proprietary hospital was found to be best, with a difference of US$127.2, compared with the public hospital, at US$45.8 (*p* < 0.001). The return earned per inpatient was also lowest for the public hospital, only 4.9%, while the medical care corporation hospital had the highest return, at 18.7% (p < 0.001) (Table 3).

**Table 3:** Medical costs

Variable	Public Hospitals		Medical Care Corporation Hospitals		Non-Profit Proprietary Hospitals		*p* ^*a*^
	(95% bootstrapped confidence interval)		(95% bootstrapped confidence interval)		(95% bootstrapped confidence interval)		
Diagnosis cost	36.2	(35.3–37.4)	23.0	(22.3–23.8)	26.3	(24.9–28)	<0.001
Ward cost	67.8	(63.6–72)	46.9	(44.8–49.2)	59.7	(54.6–65.9)	<0.001
Laboratory cost	51.8	(48.9–55.3)	56.0	(52.2–60.3)	58.3	(54.4–63.2)	<0.049
X-ray cost	7.0	(6.6–7.6)	6.7	(5.8–8.3)	7.9	(6.1–11.5)	.498
Therapeutic procedure cost	8.3	(7.5–9.6)	4.6	(4.1–5.3)	4.5	(3.8–5.7)	<0.001
Surgical cost	504.0	(500.3–508.3)	497.3	(491.6–503.1)	498.1	(492.5–503.8)	<0.094
Blood-plasma cost	1.0	(0–3.2)	0.1	(0–0.5)	0.0	(0–0)	.586
Anesthesia cost	175.2	(169.4–180.7)	95.0	(91–99.5)	107.3	(100.5–114.7)	<0.001
Special materials cost	29.0	(26.1–32.5)	18.2	(15.6–21)	35.8	(30.3–42)	<0.001
Drug cost	10.3	(9.8–11.0)	7.5	(6.8–8.4)	9.4	(8.6–10.4)	<0.001
Dispensing cost	9.1	(9.0–9.5)	5.9	(5.8–6.1)	6.8	(35.3–37.4)	<0.001
Injection service cost	2.4	(2.4–2.5)	2.5	(2.4–2.6)	2.5	(6.4–7.3)	.589
DRG fixed cost (R)	940.3	(939.6–941.1)	937.5	(929.5–941.8)	939.4	(938.3–940.4)	.125
Actual medical cost (R1)	902.7	(889.9–915.9)	764.2	(753.3–775.1)	817.1	(802.7–830.6)	<0.000
Cost difference (∆R)	45.8	(35.6–56.9)	175.3	(166.8–183.4)	127.2	(116.1–140)	<0.000
Ratio of cost difference over fixed cost (∆R/R)	4.9%		18.7%		13.5%		<0.000

Note: exchange rate: US$1 = NT$30

^a^Statistical significance was calculated by analysis of variance

The differences between hospitals in medical cost distribution of each case were statistically significant for diagnosis (US$23.0–36.2), ward (US$46.9–67.8), laboratory (US$56.0–58.3), therapeutic procedures (US$4.5–8.3), anesthesia (US$95.0–175.2), special materials (US$18.2–35.8), drugs (US$7.5–10.3), and dispensing (US$5.9–9.1) (*p* < 0.05). The differences in the cost of X-rays, surgery, and blood plasma were not statistically significant. The difference was highest for the anesthesia cost, at an average of US$80.2/case. We examined the data further to establish the causes of these cost differences (Table 3).

Table 4 shows that the public hospital used general anesthesia in 86% of cases, with local anesthesia in just 7.3% and epidural anesthesia in 6.3%. In the medical care corporation hospital and nonprofit proprietary hospital, however, spinal anesthesia was administered in more than 80% of cases (*p* < 0.001). A further examination of the correlations between the anesthesia cost and the LoS shows that the average LoS for spinal anesthesia was 1.46 days, epidural anesthesia was 2.71 days, and general anesthesia was 2.49 days (*p* < 0.001). Scheffé’s post hoc analysis shows the average LoS for local anesthesia, at 2.58 days, is longer than for spinal anesthesia, at 1.46 days (*p* < 0.001). The median number of days of hospitalization for patients receiving local, epidural, or general anesthesia was 3, while for spinal anesthesia, it was only 1 (Table 5).

**Table 4:** Anesthesia statistics (*n* = 723)

Variable	Public Hospitals		Medical Care Corporation Hospitals		Non-Profit Proprietary Hospitals		*p* ^*a*^
	*n*	%	*n*	%	*n*	%	
No. of patients	315		254		154		
Anesthesia							<0.001
Local anesthesia	23	7.3	1	0.4	0	0.0	
Spinal anesthesia	1	0.3	232	91.3	127	82.5	
Epidural anesthesia	20	6.3	0	0.0	1	0.6	
General anesthesia	271	86.0	21	8.3	26	16.9	

^a^Statistical significance was calculated using a *x* ^2^ test

**Table 5:** Anesthesia length of stay (LoS) statistics (n = 723)

Item	Variable	n	Mean	SD	*p* ^*a*^	Scheffe’s
LoS(days)					.001	① > ②
① Local anesthesia		24	2.58	.50		
② Spinal anesthesia		360	1.46	.73		
③ Epidural anesthesia		21	2.71	1.14		
④ General anesthesia		318	2.49	.97		

^a^Statistical significance was calculated using analysis of variance

## Discussion

This study investigated IHR medical cost differences among three hospitals in Taiwan under different ownership structures. The results found that LoS, the number of coding diagnoses, and medical costs differed significantly among them. Taiwan’s National Health Insurance Administration has announced rules for calculating the algebraic LoS for hospitalization in IHR cases. Some physicians will therefore try to work toward a treatment cycle of 3 days of hospitalization (admission on day 1 and a physical examination, surgery on day 2, and discharge on day 3). Others will examine patients before arranging hospitalization. Effective control of the LoS of hospitalization without affecting the quality of medical service can help to increase the hospital bed turnover rate and reduce medical care costs.

This study found that although the LoS is less than 3 days among the three different hospitals, there are still significant differences. In a study on the use of IHR resources in different DRG systems in 10 European countries, O’Reilly et al. found the LoS was 0.5–4.5 days [11]. After implementation of DRGs, the LoS was reduced by 9.0% in 1984 and 7.7% in 1985, and it continued to decrease over time. DesHarnais et al. found that after applying a PPS to the Medicare system, the average LoS in the ICU and CCU reduced significantly [12].

Additionally, after investigating the financial implications of different medical costs for IHR in hospitals under different ownership types, we confirmed the method of anesthesia appears to be associated with the LoS, and therefore with the cost to the hospital. We also discovered the medical care corporation hospital had better control over costs than the public hospital or non-profit proprietary hospital, given that the anesthesia cost showed major differences. The anesthesiologists in the public hospital preferred general anesthesia, while those in the medical care corporation hospital and nonprofit proprietary hospital tended to use spinal anesthesia. Jensen et al. [14], after analyzing 72 publications studying IHR, discovered that 60%–70% of large hospitals used general anesthesia, 10%–20% used spinal anesthesia, and 5%–15% used local anesthesia. Another study found that 95% of specialist hernia hospitals used local anesthesia [13]. The optimal method of anesthesia is still a topic of much discussion [14]. Regardless of the relative risks, the NHI reimbursement is the same. Further research is needed to understand the causes of these differences, and to improve anesthesia technique if necessary.

Furthermore, although the average number of coding diagnoses was less than two for the three hospitals in our study, the three also showed different coding diagnoses numbers for IHR; this may be a particular concern for IHR. An earlier study by O’Reilly et al. [15] found the number of diagnoses was 1.1–3.2, and the number of procedures was 1.0–4.7. Lezzoni et al. [16] stated that disease complications should be determined via correct selection of the secondary diagnosis code rather than by increasing the number of secondary diagnoses. Thus, careful monitoring of such moves needs to start from the point of implementation of the Tw-DRG-based payment system.

Finally, cost control in the medical care corporation hospital (US$764.2/case) was more efficient than in the public hospital (US$902.7/case) and nonprofit proprietary hospital (US$817.1/case). Under the Tw-DRG payment system, which is designed for cost containment and improving hospital efficiency, the financial risk of hospitals increases when there is a gap between the fixed-payment amount and the estimated medical cost, given the standard levels of reimbursement under Tw-DRGs. Hospitals must control their service expenses to remain within the amount approved under the Tw-DRGs, to reduce financial risk and to ensure the ability to deliver services. However, actual medical expenses vary even in the same Tw-DRG because of differences in practice. Therefore, hospitals need to analyze their financial risk under the Tw-DRGs so both hospital administrators and healthcare professionals are aware of the impact of their decisions.

### Limitations

Despite its careful data collection and analysis, this study had several unavoidable limitations. Previous studies have suggested that, in addition to the indices measured herein, data such as those on rates of emergency readmissions within 3 days, readmissions within 14 days, urinary tract infection, and wound infection, should be included in the analysis to enable comparisons to support the revision of operational procedures linked to Tw-DRGs. This means it is important to accurately measure medical costs. Such indices were not included as relevant factors in this study. We intend to include more flexible factors in subsequent studies.

This study is based on the NHI claims database. However, no information on patients’ lifestyles (e.g., smoking, drinking, exercise, body mass index) is available from these data. No comparison between hernia rates and other factors could be conducted in this study.

## Conclusions

This study found the medical behavior of the attending physician affects the patients’ medical resource consumption. Thus, medical costs can be minimized if the hospital can reduce both the number of days of hospitalization (via a clinical pathway strategy) and the use of medical resources, and provide the patient with adequate postoperative care. Tw-DRGs have undoubtedly led to differences among hospitals. Hospital administrators should, instead of passively waiting for improvements, establish a risk assessment system as early as possible to predict the potential medical costs of cases by using detailed diagnostic criteria prior to administration. This would enable them to grasp these costs in advance and manage and monitor them effectively. The revisions to the NHI payment system have reduced hospital income; therefore, hospitals need to implement management control systems or measures such as clinical pathways, case management, and continuous quality improvement to cost-effectively manage operational issues and sustain quality of care. Clinical pathways provide guidelines that help medical teams improve job efficiency. They also help to eliminate unnecessary tests and complications for patients, thereby improving overall patient satisfaction and reducing the length of hospital stays. This, in turn, helps increase bed turnover rates and decrease medical costs [16].

Furthermore, DRGs have been adopted worldwide as part of healthcare reimbursement systems, but they face cultural and institutional differences in different countries. Hospital administrators must, however, manage the introduction of DRGs effectively. Hospitals should understand the advantages and disadvantages of the system, and establish a strong brand to make their core services competitive. They should also improve the quality and efficiency of healthcare services to benefit the hospital, patients, and BNHI.

Finally, hospitals should by now be more aware of cost control and cost efficiency, and use this awareness to support their ongoing survival and development. As hospitals must assume full responsibility for their financial risks under the premise of self-determination, cost control will directly affect profits. This, in turn, suggests a larger challenge to hospitals’ sustainable survival. We suggest that medical institutions should educate medical personnel on the operation and impact of the Tw-DRG system. This could help nurses understand the current NHI system’s impact on quality of care and could also improve the measurement of outcomes.

## Abbreviations

IHR: Inguinal hernia repair
Tw-DRGs: Taiwan diagnosis-related groups
